# Depression follow-up monitoring with the PHQ-9: an open cluster-randomised controlled trial

**DOI:** 10.3399/BJGP.2023.0539

**Published:** 2024-06-25

**Authors:** Tony Kendrick, Christopher Dowrick, Glyn Lewis, Michael Moore, Geraldine M Leydon, Adam WA Geraghty, Gareth Griffiths, Shihua Zhu, Guiqing Lily Yao, Carl May, Mark Gabbay, Rachel Dewar-Haggart, Samantha Williams, Lien Bui, Natalie Thompson, Lauren Bridewell, Emilia Trapasso, Tasneem Patel, Molly McCarthy, Naila Khan, Helen Page, Emma Corcoran, Jane Sungmin Hahn, Molly Bird, Mekeda X Logan, Brian Chi Fung Ching, Riya Tiwari, Anna Hunt, Beth Stuart

**Affiliations:** School of Primary Care, Population Science, and Medical Education, Faculty of Medicine, University of Southampton, Aldermoor Health Centre, Southampton.; Department of Primary Care and Mental Health, Institute of Population Health, University of Liverpool, Liverpool.; Division of Psychiatry, University College London, London. Division of Psychiatry, University College London, London.; School of Primary Care, Population Science, and Medical Education, Faculty of Medicine, University of Southampton, Aldermoor Health Centre, Southampton.; School of Primary Care, Population Science, and Medical Education, Faculty of Medicine, University of Southampton, Aldermoor Health Centre, Southampton.; School of Primary Care, Population Science, and Medical Education, Faculty of Medicine, University of Southampton, Aldermoor Health Centre, Southampton.; Southampton Clinical Trials Unit, University of Southampton and University Hospital Southampton NHS Foundation Trust, Southampton.; School of Primary Care, Population Science, and Medical Education, Faculty of Medicine, University of Southampton, Aldermoor Health Centre, Southampton.; Leicester Clinical Trials Unit, University of Leicester, Leicester.; Faculty of Public Health and Policy, London School of Hygiene and Tropical Medicine, London.; Department of Primary Care and Mental Health, Institute of Population Health, University of Liverpool, Liverpool.; School of Primary Care, Population Science, and Medical Education, Faculty of Medicine, University of Southampton, Aldermoor Health Centre, Southampton.; School of Primary Care, Population Science, and Medical Education, Faculty of Medicine, University of Southampton, Aldermoor Health Centre, Southampton.; School of Primary Care, Population Science, and Medical Education, Faculty of Medicine, University of Southampton, Aldermoor Health Centre, Southampton.; School of Primary Care, Population Science, and Medical Education, Faculty of Medicine, University of Southampton, Aldermoor Health Centre, Southampton.; School of Primary Care, Population Science, and Medical Education, Faculty of Medicine, University of Southampton, Aldermoor Health Centre, Southampton.; Department of Primary Care and Mental Health, Institute of Population Health, University of Liverpool, Liverpool.; Department of Primary Care and Mental Health, Institute of Population Health, University of Liverpool, Liverpool.; Department of Primary Care and Mental Health, Institute of Population Health, University of Liverpool, Liverpool.; Department of Primary Care and Mental Health, Institute of Population Health, University of Liverpool, Liverpool.; Department of Primary Care and Mental Health, Institute of Population Health, University of Liverpool, Liverpool.; Division of Psychiatry, University College London, London. Division of Psychiatry, University College London, London.; Division of Psychiatry, University College London, London. Division of Psychiatry, University College London, London.; Division of Psychiatry, University College London, London. Division of Psychiatry, University College London, London.; Division of Psychiatry, University College London, London. Division of Psychiatry, University College London, London.; Division of Psychiatry, University College London, London. Division of Psychiatry, University College London, London.; School of Primary Care, Population Science, and Medical Education, Faculty of Medicine, University of Southampton, Aldermoor Health Centre, Southampton.; School of Primary Care, Population Science, and Medical Education, Faculty of Medicine, University of Southampton, Aldermoor Health Centre, Southampton.; Centre for Evaluation and Methods, Wolfson Institute of Population Health, Faculty of Medicine and Dentistry, Queen Mary University of London, London.

**Keywords:** primary health care, mental health, mood disorders, depression, patient reported outcome measures

## Abstract

**Background:**

Outcome monitoring of depression treatment is recommended but there is a lack of evidence on patient benefit in primary care.

**Aim:**

To test monitoring depression using the Patient Health Questionnaire (PHQ-9) with patient feedback.

**Design and setting:**

An open cluster-randomised controlled trial was undertaken in 141 group practices.

**Method:**

Adults with new depressive episodes were recruited through record searches and opportunistically. The exclusion criteria were as follows: dementia; psychosis; substance misuse; and suicide risk. The PHQ-9 was administered soon after diagnosis, and 10–35 days later. The primary outcome was the Beck Depression Inventory (BDI-II) score at 12 weeks. The secondary outcomes were as follows: BDI-II at 26 weeks; Work and Social Adjustment Scale (WSAS) and EuroQol EQ-5D-5L quality of life at 12 and 26 weeks; antidepressant treatment; mental health and social service contacts; adverse events, and Medical Interview Satisfaction Scale (MISS) over 26 weeks.

**Results:**

In total, 302 patients were recruited to the intervention arm and 227 to the controls. At 12 weeks, 254 (84.1%) and 199 (87.7%) were followed-up, respectively. Only 40.9% of patients in the intervention had a GP follow-up PHQ-9 recorded. There was no significant difference in BDI-II score at 12 weeks (mean difference −0.46; 95% confidence interval [CI] = −2.16 to 1.26; adjusted for baseline depression, baseline anxiety, sociodemographic factors, and clustering by practice). EQ-5D-5L quality-of-life scores were higher in the intervention arm at 26 weeks (adjusted mean difference 0.053; 95% CI = 0.013 to 0.093. A clinically significant difference in depression at 26 weeks could not be ruled out. No significant differences were found in social functioning, adverse events, or satisfaction. In a per-protocol analysis, antidepressant use and mental health contacts were significantly greater in patients in the intervention arm with a recorded follow-up PHQ-9 (*P* = 0.025 and *P* = 0.010, respectively).

**Conclusion:**

No evidence was found of improved depression outcome at 12 weeks from monitoring. The findings of possible benefits over 26 weeks warrant replication, investigating possible mechanisms, preferably with automated delivery of monitoring and more instructive feedback.

## Introduction

Guidelines on the management of depression in adults recommend practitioners consider using validated patient-reported outcome measures (PROMs) to inform treatment at diagnosis and follow-up of people with depression,^1^^–^^4^ but there is insufficient evidence that they improve depression management and outcomes for patients in primary care.^5^^,^^6^

Relatively few studies of PROMs for depression have been conducted in primary care, and there is almost a complete lack of evidence on important outcomes including social functioning, patient satisfaction, quality of life, cost-effectiveness, and possible adverse effects.^5^^,^^6^

The aim of the study was to answer the research question: what is the effectiveness of assessing primary care patients with depression or low mood after diagnosis and again at follow-up 10–35 days later, using the Patient Health Questionnaire (PHQ-9) as a PROM, giving practitioners guidance on assessment and feedback to patients on their progress?

## Method

### Design and setting

A parallel group open cluster-randomised superiority trial was set in 141 group general practices in England and Wales. A cluster-randomised design was chosen based on a significant risk of contamination between arms identified through qualitative interviews with GPs in a prior feasibility trial; that is, that it would be difficult to forget and avoid using the PHQ-9 questions when treating a control patient in an individually randomised trial.

**Table:** How this fits in

Follow-up monitoring of people with depression, using patient-reported outcome measures (PROMs), is recommended but evidence of benefit in primary care is lacking. Monitoring patients’ progress with the Patient Health Questionnaire (PHQ-9) produced no benefit in terms of depressive symptoms at 12 weeks follow-up, but at 26 weeks a significant difference in depression could not be ruled out, and patients’ quality of life was significantly improved (*P* = 0.01). Only 40.9% of patients in the intervention arm had a follow-up PHQ-9 recorded in the GP records. Further research should test PROMS that measure anxiety as well as depression, which are automatically delivered and integrated into patients’ records, and produce specific treatment recommendations.

### Randomisation

Randomisation was carried out remotely by a clinical trials unit statistician using computerised sequencing, with minimisation for three factors: recruiting centres; small or large practices (dichotomised around 8000 patients); and urban or rural locations by local authority classification.

### Inclusion and exclusion criteria

Adults with new episodes of depression were recruited mainly through frequent practice record searches, but also opportunistically in consultations. Exclusion criteria were as follows: existing treatment for depression; dementia; psychosis; substance misuse; or suicide risk.

### Intervention

The PHQ-9^7^ was administered by a researcher as soon as possible after recruitment (within 2 weeks), and the GP was asked to repeat the PHQ-9 at a follow-up consultation 10–35 days later. Patients were given written feedback on their PHQ-9 scores and potential treatments to discuss with their GPs. The GPs were given 2 hours online training in interpreting PHQ-9 scores and taking them into account in management (Supplementary Information S1). They were tested on their understanding of the trial processes together with the strengths and limitations of the PHQ-9, and how it might be used in practice (Supplementary Information S2). Use of the PHQ-9 in practice was modelled by one of the co-principal investigators (CD), with a simulated patient, in videos representing the first and second follow-up consultations for depression with a practitioner in the study (Supplementary Information S3).

### Outcomes

The primary outcome was depression on the Beck Depression Inventory (BDI-II)^8^ at 12 weeks. The secondary outcomes at 26 weeks were as follows: BDI-II scores; social functioning (Work and Social Adjustment Scale [WSAS]);^9^ quality of life (EuroQol EQ-5D-5L);^10^ patient satisfaction (Medical Interview Satisfaction Scale [MISS]);^11^ antidepressant treatment; mental health and social service contacts; and adverse events.

### Blinding

Blinding of participants to allocation was impossible given the pragmatic cluster randomised design, but self-report outcome measures were used to prevent observer bias, and analysis was blind to allocation.

### Sample size calculation

A baseline mean BDI-II score of 24.0 was assumed with a standard deviation (SD) of 10.0 (derived from a feasibility trial),^12^ and mean scores of 14.0 and 17.0 at 12 weeks in the intervention and control groups, respectively. An effect size of 0.3 SD represented the minimum clinically important difference (MCID) on the BDI-II.^13^ At 5% significance, for 90% power, 235 patients were needed to be analysed per group. An intracluster correlation coefficient (ICC) of 0.03 was assumed (from the feasibility trial)^12^ and mean cluster size of six, which gave a design effect of 1.15, giving 270 per group. With 20% loss to follow-up, the target was 676 patients recruited from 113 practices.

The target was subsequently (10 June 2021) revised on finding a correlation of greater than *P* = 0.5 between baseline and follow-up for the primary outcome, meaning only 222 patients were needed to be analysed per group and total target of 554 recruited.

### Analysis

A detailed statistical analysis plan was drawn up before analysis of the results (Supplementary Information S4). Differences between arms in depressive symptoms, social functioning, and quality of life at 12 and 26-weeks follow-up were analysed using linear mixed models, adjusting for baseline values; baseline anxiety (measured using the Generalised Anxiety Disorder-7item [GAD-7]);^14^ sociodemographic factors, past history of depression, and clustering including a random effect for practice. Patient satisfaction was compared between arms over the 26-week period.

Differences between arms in the process of care for depression were also analysed from practice medical record data over 26 weeks including PHQ-9s recorded, antidepressant prescribing, and mental health and social service contacts.

### Suicide risk

If patients scored other than 0 on suicide or self-harm questions on the BDI-II or PHQ-9 at screening, baseline, or follow-up, or indicated suicidal ideas in other ways, a standard operating procedure (SOP) was implemented, requiring further assessment using the P4 screener suicide risk assessment.^15^ Based on the patients’ responses, the risk of suicide was categorised as minimal, lower, or higher, and the GP was informed immediately. Care of all patients remained the responsibility of participating GPs, as in usual practice.

More information on the methods can be found in the published protocol.^16^

## Results

### Recruitment of practices

The study aimed to recruit 113 practices between November 2018 and August 2019. Owing to slow recruitment of patients, the recruitment process had to continue much longer than planned (made worse by the COVID-19 pandemic), eventually a total of 189 was reached by December 2021. Then 48 withdrew before recruiting patients (24 in each arm), leaving 141: 72 intervention and 69 control-arm practices. Minimisation ensured practice characteristics were balanced by arm (Table 1).

**Table 1.** Cluster level (participating general practice) characteristics at baseline

**Characteristic**	**Intervention**	**Control**	**Total**
**Centre**			
Southampton	25	27	52
Liverpool	31	28	59
London	40	38	78

**List size**			
Small	34	32	66
Large	62	61	123

**Location**			
Urban	77	77	154
Rural	19	16	35

### Recruitment of patients

**Figure 1.**
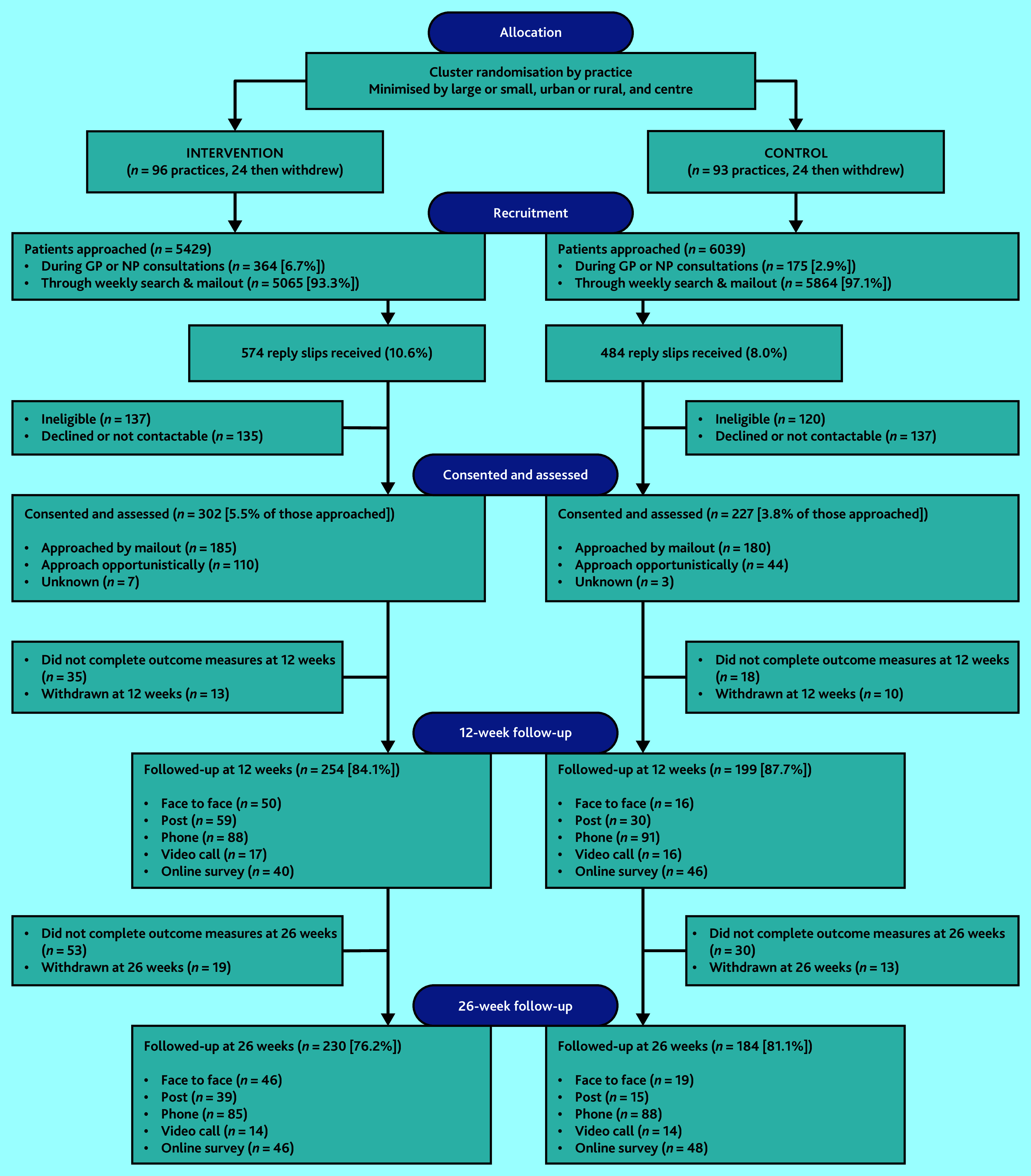
CONSORT diagram. NP = nurse practitioner. NB: 58 intervention arm patients who missed follow-up at 12 weeks returned to follow-up at 26 weeks.

Of 11 468 patients approached in consultations or through mailed invitations, 1058 (9.2%) returned reply slips; 574/5429 (10.6%) patients approached in the intervention arm and 484/6039 (8.0%) patients approached in the control (Figure 1). After exclusion of patients declining participation, ineligible, or uncontactable, 529 were assessed at baseline: 302/5429 (5.5%) in the intervention arm and 227/6039 (3.8%) in the control, between January 2019 and March 2022. The ratio of intervention to control arm patients was therefore 1.3 to 1.

### Follow-up of patients

Of 529 patients recruited, 453 (85.6%) were followed up at 12 weeks: 254/302 in the intervention arm (84.1%) and 199/227 in the controls (87.7%). At 26 weeks, 414 (78.3%) were followed-up: 230/302 in the intervention arm (76.2%) and 184/227 (81.1%) in the controls (Figure 1). Medical records data were collected for 259 (85.8%) patients in the intervention arm and 201 (88.5%) controls.

### Baseline characteristics

Mean baseline BDI-II score was higher in the intervention arm at 24.1 (SD 8.89) compared with 22.4 (SD 9.52) in the control (Table 2). Baseline anxiety and quality of life were also worse in the intervention arm. Patients in the control arm were more likely to have had two or more previous depressive episodes. Sociodemographic characteristics were relatively well-balanced, apart from patients in the control arm being more likely to have no dependants (Table 2).

**Table 2.** Participating patient characteristics at baseline

**Characteristic**	**Intervention (*n* = 302)**	**Control (*n* = 227)**	**Total (*n* = 529)**
Mean baseline depression score on the BDI-II (SD)	24.1 (8.89)	22.4 (9.52)	23.4 (9.2)

Mean baseline anxiety score on the GAD-7 (SD)	12.8 (5.31)	11.8 (5.58)	12.4 (5.45)

Mean baseline quality-of-life score on the EQ-5D-5L (SD)	0.659 (0.232)	0.667 (0.226)	0.663 (0.230)

Mean duration of depression in years (SD)	3.4 (5.13)	2.6 (5.56)	3.1 (5.33)

Previous depression, *n* (%)			
None	87 (28.8)	46 (20.3)	133 (25.1)
Once before	79 (26.2)	62 (27.3)	141 (26.7)
Twice or more before	135 (44.7)	119 (52.4)	254 (48.0)

Female, *n* (%) (Self-declared gender)	192 (63.6)	136 (59.9)	328 (62.0)

Mean age in years at baseline (SD)	45.2 (15.94)	45.0 (17.17)	45.1 (16.46)

Ethnic group, *n* (%)			
White	255 (84.4)	193 (85.0)	448 (84.9)
Black Caribbean	1 (0.3)	3 (1.3)	4 (0.8)
Black African	3 (1.0)	4 (1.8)	7 (1.3)
Black other	2 (0.7)	0 (0.0)	2 (0.4)
Indian	13 (4.3)	4 (1.8)	17 (3.2)
Pakistani	6 (2.0)	4 (1.8)	10 (1.9)
Bangladeshi	0 (0.0)	1 (0.4)	1 (0.2)
Chinese	4 (1.3)	3 (1.3)	7 (1.3)
Other Asian group	5 (1.7)	3 (1.3)	8 (1.5)
Other ethnic group	12 (4.0)	12 (5.3)	24 (4.5)

Socioeconomic position, *n* (%)			
Full-time work	140 (46.4)	113 (49.8)	253 (47.8)
Part-time work	55 (18.2)	28 (12.3)	83 (15.7)
Permanently sick or disabled	5 (1.7)	6 (2.6)	11 (2.1)
Unemployed	36 (11.9)	18 (7.9)	54 (10.2)
Retired	33 (10.9)	31 (13.7)	64 (12.1)
Student	8 (2.6)	12 (5.3)	20 (3.8)
Homemaker	5 (1.7)	4 (1.8)	9 (1.7)
Voluntary work	6 (2.0)	4 (1.8)	10 (1.9)
Other	14 (4.6)	11 (4.8)	25 (4.7)

Accommodation, *n* (%)			
Owner-occupied	142 (47.0)	106 (46.7)	248 (46.9)
Council or housing association	39 (12.9)	20 (8.8)	59 (11.2)
Private rental	71 (23.5)	57 (25.1)	128 (24.2)
Job related	2 (0.7)	1 (0.4)	3 (0.6)
Lives with parents	40 (13.2)	34 (15.0)	74 (14.0)
Other	8 (2.6)	9 (4.0)	17 (3.2)

Highest educational qualification,^a^ *n* (%)			
None	26 (8.6)	20 (8.8)	46 (8.7)
CSE or NVQ Level 1	22 (7.3)	3 (1.3)	25 (4.7)
GCSE or O Level	49 (16.2)	33 (14.5)	82 (15.5)
A Level or BTEC	54 (17.9)	41 (18.1)	95 (18.0)
HNC, HND or City & Guilds	24 (8.0)	16 (7.0)	40 (7.6)
Degree or higher degree	111 (36.8)	90 (39.6)	201 (38.0)
Vocational qualification	8 (2.6)	14 (6.2)	22 (4.2)
Other	5 (1.7)	8 (3.5)	13 (2.5)

Marital status, *n* (%)			
Married	119 (39.4)	83 (36.6)	202 (38.2)
Cohabiting	26 (8.6)	26 (11.5)	52 (9.8)
Widowed	10 (3.3)	10 (4.4)	20 (3.8)
Separated	11 (3.6)	6 (2.6)	17 (3.2)
Divorced	25 (8.3)	13 (5.7)	38 (7.2)
Single	111 (36.8)	89 (39.2)	200 (37.8)

Number of dependants in the household, *n* (%)			
None	174 (57.6)	151 (66.5)	325 (61.4)
1	43 (14.2)	34 (15.0)	77 (14.6)
2	56 (18.5)	26 (11.5)	82 (15.5)
3	15 (5.0)	11 (4.8)	26 (4.9)
4	9 (3.0)	3 (1.3)	12 (2.3)
5	2 (0.7)	0 (0.0)	2 (0.4)

a *CSE is the Certificate of Secondary Education, a qualification in a specific subject formerly taken by school students aged 14–16 years, at a level below O (Ordinary) level. Both the CSE and O level were replaced in 1988 by the GCSE, or General Certificate of Secondary Education. NVQ Level 1 is the first level National Vocational Qualification, a work-based job-specific qualification. A Level is the Advanced secondary education qualification in a specific subject taken by school students aged 17–19 years. BTEC is the Business and Technology Education Council certificate work-based vocational qualification taken after secondary school for aged >16 years. HNC (Higher National Certificate), HND (Higher National Diploma), and City & Guilds are more advanced vocational qualifications. BDI-II = Beck Depression Inventory, Second Edition.* GAD-7 = Generalised Anxiety Disorder assessment. SD = standard deviation. *NB: Where the values do not add up to the total n followed up, it is because some patients did not answer all questions and therefore there are missing values for some items.*

### Primary outcome

**Figure 2.**
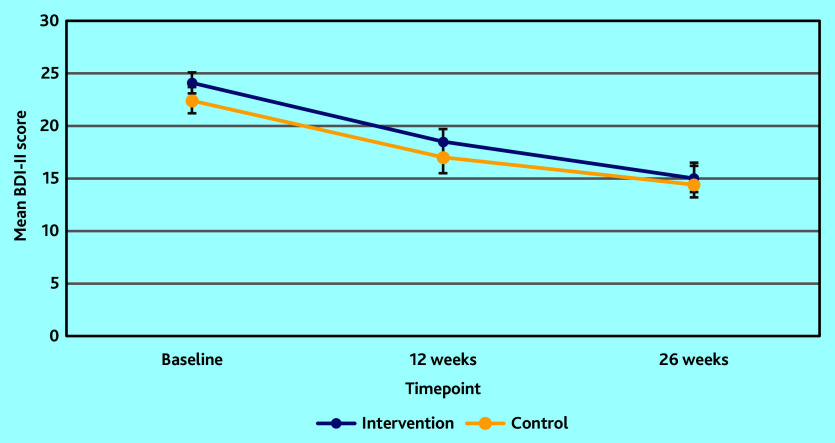
Mean BDI-II depression scores at baseline, 12 weeks, and 26 weeks follow-up.

At 12 weeks follow-up, the mean BDI-II score was 18.5 (SD 10.2) in the intervention arm and 16.9 (10.3) in the control (Table 3 and Figure 2). The adjusted mean score was slightly lower in the intervention arm, but not statistically significant (adjusted mean difference −0.46; 95% confidence interval (CI) = −2.16 to 1.26; *P* = 0.60).

**Table 3.** Primary and secondary outcomes at baseline, 12 weeks, and 26 weeks follow-up

	**Baseline**		**12 weeks**				**26 weeks**	

	***n***	**Mean score (SD)**	***n***	**Mean score (SD)**	**Mean adjusted difference^a^ (95% CI); *P*-value**	***n***	**Mean score (SD)**	**Mean adjusted difference^a^ (95% CI); *P*-value**
**Depression (BDI-II score)**								
Intervention	302	24.1 (8.96)	252	18.5 (10.17)	−0.46 (−2.16 to 1.26); *P* = 0.602	226	15.1 (10.84)	−1.63 (−3.48 to 0.21); *P* = 0.082
Control	227	22.4 (9.52)	195	16.9 (10.30)	REF	184	14.7 (10.65)	REF

**Social functioning (WSAS score)**								
Intervention	302	17.3 (9.94)	237	14.7 (9.54)	0.48 (−1.03 to 2.00); *P* = 0.531	212	11.6 (9.59)	1.34 (−3.20 to 0.53); *P* = 0.160
Control	227	16.6 (10.06)	195	13.2 (9.90)	REF	183	12.0 (9.99)	REF

**Quality of Life (EQ-5D-5L score)**								
Intervention	302	0.659 (0.232)	256	0.694 (0.236)	−0.002 (−0.0412 to 0.0372) *P* = 0.94	221	0.718 (0.249)	0.053 (0.013 to 0.093); *P* = 0.01
Control	226	0.667 (0.226)	197	0.708 (0.213)	REF	183	0.696 (0.225)	REF

**Satisfaction with care (MISS total score)**								
Intervention	302	N/A		N/A		217	121.8 (27.37)	5.39 (−1.39 to 12.16); *P* = 0.119
Control	227	N/A		N/A		176	116.0 (26.75)	REF

a *Adjusted for baseline value, baseline anxiety (GAD-7 score), sociodemographics, past history of depression, and practice as a random effect. BDI-II = Beck Depression Inventory, Second Edition.* GAD-7 = Generalised Anxiety Disorder questionnaire. *MISS = Medical Interview Satisfaction Scale. REF = reference value.* WSAS *= Work and Social Adjustment Scale.*

b *One patient withdrew immediately after enrolment and did not complete an EQ-5D-5L at baseline.*

At 26 weeks both groups improved further on the BDI-II (Table 3 and Figure 2). The score was slightly lower in the intervention arm, but not significantly (adjusted mean difference −1.63; 95% CI = −3.48 to 0.21; *P* = 0.08). The 95% CI included a difference favouring the intervention by more than 3.0 points on the BDI-II so a clinically important difference in depression at 26 weeks could not be excluded.

As a sensitivity analysis, the primary outcome was re-analysed using a multiple imputation model, including the baseline value, clustering by practice, and all covariates included in the model. The inferences at 12 and 26 weeks were unchanged (adjusted mean difference at 12 weeks −0.18; 95% CI = −1.82 to 1.45; *P* = 0.83, and at 26 weeks −0.93; 95% CI = −2.69 to 0.83; *P* = 0.30).

### Secondary outcomes

A similar pattern was seen for social functioning at 12 and 26 weeks, with scores improving between baseline and 12 weeks, and further by 26 weeks, but no significant difference between arms (Table 3).

**Figure 3.**
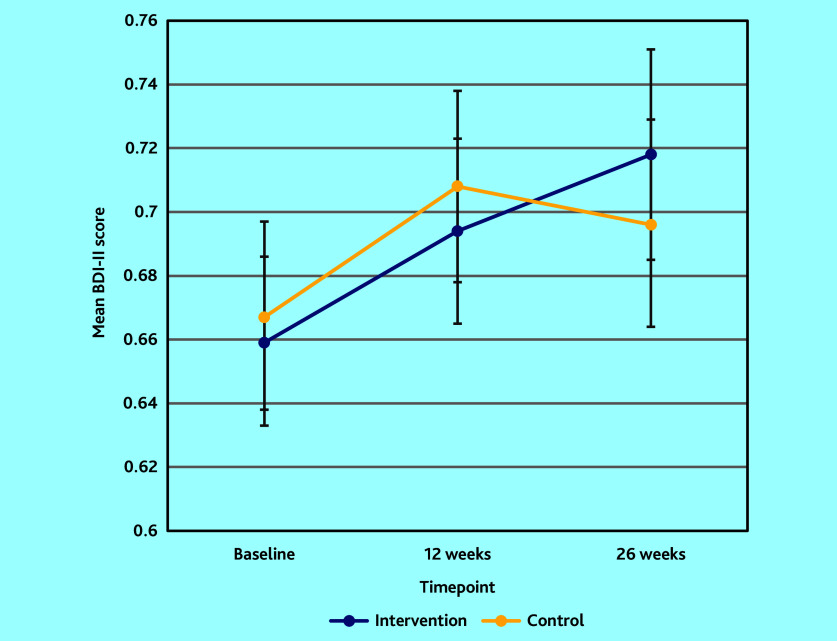
Mean EQ-5D-5L quality-of-life scores at baseline, 12 weeks, and 26 weeks follow-up.

Quality of life improved in both arms between baseline and 12 weeks, then improved further in the intervention arm, but went down slightly in the control (Table 3 and Figure 3). The difference between arms was not statistically significant at 12 weeks but was significant at 26 weeks, favouring the intervention (adjusted mean difference 0.053; 95% CI = 0.013 to 0.093; *P* = 0.01).

Patient ratings in the two arms were similar at baseline on the EQ-5D-5L subscales for mobility, self-care, and pain or discomfort, and remained so (Supplementary Table S1). Slightly more patients in the intervention declared severe or extreme problems for anxiety and depression at baseline (23.5% versus 19.5%). At 26 weeks follow-up, the proportions declaring no problem with anxiety and depression were 22.6% in the intervention arm versus 13.5% in the control. Improvement in the anxiety and depression dimension therefore explained the overall greater improvement in scores in the intervention arm.

Total scores for satisfaction with care looking back over 26 weeks were very similar between arms (Table 3). The mean score for the intervention arm was 121.8 and 116.0 for the control arm. The same was found for all four satisfaction subscales (Distress–relief, Communication–comfort, Rapport, and Compliance–intent).

### Post-hoc analysis of 50% improvement, and remission, at 26 weeks

A post-hoc analysis was conducted of categorical improvements in BDI-II scores at 26 weeks, to further investigate differences in depression, given the wide CIs around the mean difference, and because of the difference that was found in proportions of patients reporting no anxiety and depression on the EQ-5D-5L at 26 weeks (Supplementary Table S1). The proportions in each arm who improved by ≥50% on the BDI-II, were compared with the proportions who scored >13 at baseline (the threshold for ‘caseness’) and subsequently remitted to ≤13 by 26 weeks.

The proportions of patients improving by ≥50% were not significantly different (102/226 intervention [45.1%] versus 69/185 [37.3%] controls; OR 1.53; 95% CI = 0.92 to 2.56; *P* = 0.10), but the proportion of patients remitting in the intervention arm was significantly greater (100/201 [49.8%] versus 59/148 [39.9%]; OR 2.18; 95% CI = 1.12 to 4.24; *P* = 0.02).

### Post-hoc per-protocol analysis of depression outcome

In the intervention arm 190 out of 259 patients (73.4%) had PHQ-9s recorded in the medical record, and in the control arm 35 out of 201 patients (17.4%). However, around half of those recorded were the baseline PHQ-9s carried out by the researchers. Only 124 patients had recorded PHQ-9s carried out by their GPs during follow-up: 106 in the intervention arm (40.9%) and 18 in the control arm (8.9%).

Post-hoc, GP compliance was defined with the protocol in the intervention arm as carrying out and recording a follow-up PHQ-9, and in the control arm with not carrying out and recording one. On that basis, a post-hoc per-protocol analysis of depression outcome was carried out for the 106 intervention participants with a recorded follow-up PHQ-9, compared with that for the 209 control participants without a recorded follow-up PHQ-9. At 12 weeks the fully adjusted difference in BDI-II score was −1.57 points (95% CI = −3.47 to 0.35; *P* = 0.108) and at 26 weeks −1.08 (95% CI = −3.40 to 1.24; *P* = 0.361), so there were no significant differences in depression symptom counts at either point.

### Use of antidepressants

Medical records data were obtained for 259 intervention arm patients (85.8%) and 201 controls (88.5%). Of these 174 (67.2%) and 112 (55.7%), respectively, had antidepressant prescriptions recorded over 26 weeks, but the difference between arms was not significant (odds ratio (OR) 1.83; 95% CI = 0.96 to 3.48; *P* = 0.07, adjusted for baseline depression, baseline anxiety, baseline antidepressant use, sociodemographics, and practice).

A post-hoc per-protocol analysis found that of the 106 patients in the intervention with a recorded follow-up PHQ-9, 71 (67.0%) received a prescription for antidepressants, compared with 102/183 (55.7%) of the 209 controls with no recorded follow-up PHQ-9. The adjusted OR was 2.80 (95% CI = 1.14 to 6.88; *P* = 0.025), showing significantly more antidepressant prescribing in those with a recorded follow-up PHQ-9 administered by the GP.

### Contact with mental health and social services

In their records, 90 out of 259 patients in the intervention arm (34.7%) and 68 out of 201 controls (33.8%) had contacts over 26 weeks with mental health and social services (mental health nurse, counsellor, psychologist, psychiatrist, and social workers), which were not significantly different between arms (adjusted OR 1.37; 95% CI = 0.71 to 2.63; *P* = 0.342).

In a post-hoc per-protocol analysis, 48 (45.3%) of the 106 patients in the intervention arm with a recorded follow-up PHQ-9 had a mental health service contact, compared with 57/183 (31.1%) of the controls with no follow-up PHQ-9. The adjusted OR was 3.96 (95% CI = 1.38 to 11.34; *P* = 0.010) showing significantly more mental health contacts for those with a recorded follow-up PHQ-9 administered by the GP.

### Adverse events

There were two serious adverse events. One patient in the control arm reported suicidal ideas; they were assessed by the trial principal investigator and found to be at higher risk, and the GP was informed immediately. The patient was referred to a community mental health team (CMHT) for immediate assessment and withdrawn from the study. One patient in the intervention arm was hospitalised with COVID-19 and ketoacidosis: a severe event, but not related to the trial.

The suicidal ideation SOP was triggered 318 times, 180 times for patients in the intervention arm patients, and 138 times for controls, in proportion to patient numbers in each arm. Altogether, 267 (146 intervention, 121 control) were rated ‘minimal risk’, 38 (25 intervention, 13 control) ‘lower risk’, and 13 (nine intervention, four control) ‘higher risk’. In four cases (two intervention and two control) participants were withdrawn from the study.

## Discussion

### Summary

No significant difference was found between the intervention and control arms in the primary outcome, which was depression on the BDI-II at 12 weeks. However, it was not possible to rule out a clinically significant benefit at 26 weeks, given the upper limit of the 95% CI included the MCID of 3.0 points.^13^ Evidence was found of benefit in a categorical analysis of remission of depression to a BDI-II <13 at 26 weeks, but this was a post-hoc analysis, and there was no significant difference in a similar analysis of the proportions of patients with a 50% improvement in depression.

There were no significant differences found in social functioning and satisfaction with care, although the differences found tended to favour the intervention. Quality-of-life scores were, however, significantly higher in the intervention arm at 26 weeks. The better quality-of-life score was owing to a greater proportion of patients in the intervention arm reporting no anxiety and depression. Anxiety symptoms were not measured specifically at follow-up, but it may be that some patients were reassured to see their depression was improving, and therefore felt less anxious.

Overall, more patients in the intervention arm than controls had recorded antidepressant prescriptions over 26 weeks (67.4% versus 55.7%), but this difference was not statistically significant. There was no overall difference in mental health and social service contacts either, with one-third of patients in both arms having at least one. However, in post-hoc per-protocol analyses, including only those patients in the intervention arm who had follow-up PHQ-9s administered and recorded by their GPs, there was significantly greater antidepressant prescribing and contact with mental health services than among controls with no follow-up monitoring.

### Strengths and limitations

A strength of the study is that its design was informed by a feasibility trial,^12^ which led to choosing the cluster design, avoiding contamination between arms in applying the intervention, and optimising adherence to study procedures in practices. However, a cluster randomised design increases the risk of selection bias among practitioners deciding whether to approach patients opportunistically in consultations. More than twice as many patients in the intervention arm than control arm were recruited opportunistically, and overall the ratio of patients randomised was 1.3 to 1, which may have reflected lower motivation to take part on the part of patients in the control arm, who were offered only usual care. Selection bias may explain higher baseline depression and anxiety scores, and lower quality of life, in the intervention arm, although the two arms were relatively well-balanced in terms of patient demographics, and analyses were adjusted for baseline differences.

Participating practitioners were trained in both the use of the PHQ-9 and treatment choices related to severity scores, while considering contextual factors. The amount of training was limited to 2 hours but was considered an amount feasible to offer at scale.

Recruitment to the trial was challenging, particularly during the COVID-19 pandemic, when practices had significant extra pressures. The revised target of 554 patients was not quite achieved, by 25, but the follow-up rate of 85.6% was better than predicted and primary outcome data were gathered on sufficient participants to answer the main research question with precision, so the result for the primary outcome may be regarded as robustly negative. It is possible, however, that there was a difference in depression at 26 weeks, which was missed owing to lacking power at that point.

It was not possible to blind participants and researchers given the pragmatic cluster design, but self-report outcome measures avoided possible observer bias, and the statistical analyses were all carried out blind to allocation.

Delivering the intervention was challenging, and not as it would be in routine practice. Practitioners could not administer the PHQ-9 when patients first presented with depression, because patients had to be given information about the study and at least 24 hours to consider taking part before consenting. This was a requirement of the NHS Research Ethics Committee. To avoid asking the GP to bring the patient back to administer the first PHQ-9, the researcher administered it at baseline assessment instead. Treatment could therefore have started at the initial consultation before the baseline score could be taken into account.

As only 73.4% of patients in the intervention arm had PHQ-9s recorded in their records, the GPs obviously did not record their scores routinely, since it is known 100% had PHQ-9s administered by the researchers at baseline, and these were all communicated to the practices. Only 40.9% of patients in the intervention arm had follow-up, GP-administered PHQ-9s recorded, although the actual numbers of follow-up PHQ-9s carried out may well have been higher. The GPs in the intervention arm were asked to administer follow-up PHQ-9s with all their participating patients but did not insist that they recorded the follow-up PHQ-9s, which is a limitation of the study. Effectively, instituting a policy of monitoring using the PHQ-9 was tested, which was known would not necessarily be carried out per protocol, which would likely be the case to a greater extent in routine practice.

The resources were not available to collect detailed information in real-time of individual GPs’ patient treatment plans and whether they were changed following PHQ-9 assessment at follow-up. However, the post-hoc per-protocol analyses, indicating that significantly increased antidepressant prescribing, and mental health service contacts were associated with carrying out and recording follow-up PHQ-9s, suggested that the GPs may have increased antidepressant treatment and referrals to specialist services on finding less than desired improvements in scores at follow-up.

A smaller proportion (17.4%) of patients in the control arm also had at least one PHQ-9 recorded, despite the fact practitioners in the control arm were asked not to use them. These may have been administered outside practices in psychology services, or by temporary practitioners within practices. However, this was a relatively low level of use, so there was good differentiation between the arms, and the pre-specified analyses were conducted on an intention-to-treat basis.

There were relatively few exclusion criteria, tending to increase the heterogeneity of the sample and generalisability of the findings. The computer codes used to identify patients through the records searches included symptom codes (for example, ‘low mood’) in addition to specific diagnoses (for example, ‘depressive disorder’), to avoid missing patients not given a specific diagnosis.

However, there was a relatively large drop-off from the 11 468 patients approached to take part down to the 529 who eventually consented and were enrolled in the study, only 5.5% of those approached in the intervention arm, and 3.8% in the control.

### Comparison with previous literature

The findings are consistent with previous trials that have mostly shown no benefit for depression outcome. Only one trial found a reduction in depression,^17^ but no changes in management to explain the benefit.^18^ Two others found changes in management but not outcomes*.*^19^^,^^20^ The most recent found no difference in depression, but reduced anxiety at 8 weeks, and improved functioning at 24 weeks follow-up.^21^

Evidence of benefit from PROMs has been found in psychological therapy settings including improved outcome,^22^ and making therapy more efficient.^23^ However, in psychological services PROMs are given multiple times during therapy, and facilitate adjustment of treatment. With only 1–2 PHQ-9s given in the present study, the information available to GPs was more restricted. PROMs used in monitoring progress in psychological services are also more extensive than the PHQ-9 alone, and the implications of results for therapy are discussed with supervisors between sessions.^22^^,^^23^ Finally, psychological services offer a range of evidence-based treatments, whereas GP treatment is largely antidepressants alone, and may be less effective in changing depression outcome.^1^

### Implications for research and practice

The absence of evidence for improvement in the outcome of depression from studies of follow-up monitoring with PROMs in primary care suggests that guidelines that recommend their use^1^^–^^4^ should continue to make them discretionary rather than mandatory, at least outside psychological therapy settings, where there is good evidence of benefit. Monitoring patients who like to see improvement in their scores is justifiable, as it may improve their quality of life. The post-hoc analyses also suggest that conducting and recording follow-up PHQ-9s may lead to greater antidepressant prescribing and referrals to mental health services. However, their use is not without cost, in terms of the time taken, even though they are relatively cheap. The cost-effectiveness of using the PHQ-9 in this study will be reported separately.

In addition, continuing to recommend outcome monitoring with PROMs may be justified based on providing greater transparency to health service funders and the public about the management of depression and patients’ responses to particular treatments.

Future research on depression monitoring in primary care should improve the delivery of monitoring and test PROMs, which cover anxiety and social functioning as well as depression. PROMS should be completed remotely between consultations; facilitated by automated analysis and feedback of the results to practitioners and patients; and specific recommendations for treatment should be delivered. Practitioners interpreting PROM results will still need to consider the circumstances surrounding individuals’ histories of depression, and responses to treatments.

## Data

Our research ethics permission is for use of the data by the research team only. Applications to use the data need to be in the form of a peer-reviewed protocol, and will be considered by the three lead investigators, Tony Kendrick, Glyn Lewis and Mark Gabbay, in the first instance, before a decision on data sharing will be taken by the rest of the co-authors. Qualitative data will not be shared, due to the relatively high risk of breaches of confidentiality arising from the nature of qualitative interviews, which are difficult to make completely anonymous.

## Patient and public involvement

The authors are grateful for the patient and public involvement. See Supplementary Information S6 for details.

## Trial registration number

International Standard Randomised Controlled Trial Number (ISRCTN) no: 17299295.
